# Contributions to the History of Malaria

**Published:** 1843-11-25

**Authors:** 


### CONTRIBUTIONS TO THE HISTORY OF MALARIA.

[Communicated by Professor Dunglison.]

*Philadelphia, Nov.* 20, 1843.

My Dear Sir,—The following communications relate to a terrestrial or geological phenomenon, always of deep interest to the profession; and I have no doubt, that the respectable and intelligent writers will pardon me for making them public.

I am, my dear sir,
Very truly yours,
Robley Dunglison.

Dr. Clymer.

*Philadelphia, January 18th*, 1843.

Sir,—Your remarks on the subject of Malaria, in a lecture] a few afternoons since, bring to my mind, in a manner more forcible than they were ever presented to it before, some circumstances which came under my observation in the year 1841. Late in the month of July of that year, I was put in medical charge of a party of about forty persons, who were about making a journey from Fort Van Couver, on the Columbia river, to the mouth of the Rio Sacramiento, in Upper California. After we had proceeded about one hundred and thirty miles, and had reached the banks of the Wallamette, opposite the American Missionary settlement, counter-orders reached us, and we were detained, encamped immediately on the bank of this river for a month or nearly so. During this time, almost the whole party were attacked with intermittent fever, generally assuming the tertian type; and, in a few instances, the quotidian. I was called also to several cases in the neighbouring country. Through this region runs the Wallamette, a rapid stream two hundred yards wide. The shores are thickly covered with a fine growth of pine and other trees, and the undergrowth is rich and luxuriant. A very short distance from the river, the country is prairie (hill and dale), with occasional groves and narrow strips of oak; and here there is no undergrowth (except a high grass, which does not rot in summer, but is perfectly cured, there being no rain) the surface being as clean as if attended to with the most constant care. The climate is very peculiar. In midsummer, when we were there, the thermometer frequently ranged from 80 to 85° F. in the shade, and, notwithstanding this great degree of diurnal heat, there was often frost; and sometimes ice an eighth of an inch in thickness at night. The whole river-region was enveloped in a thick fog every morning, so thick sometimes, that it was impossible to see a man twenty steps off. The weather was perfectly clear almost the whole time; there not being rain, enough to wet to the skin during the month. The soil is generally light, and gravelly. We lived in tents *immediately* on the bank of the river, sleeping on the ground from which we were only separated by a blanket or bearskin. The missionaries and other whites, living on the river in houses, were affected with the malady, but not to so great an extent as ourselves; and numbers of the Indians had it also. It was equally prevalent on the *upper* part of the Columbia, where the whites in the employ of the Hudson's Bay Co., and the Indian inhabitants suffered largely. But, in the settlement at the mouth of the Columbia, near the sea, I was informed, that the inhabitants were entirely free from it, as were those few settlers, who lived in the upper country at a distance from the large water-courses. In some districts, the Indians have died by hundreds. This is probably, in part at least, owing to their mode of treatment, which consists in heating themselves in a vapour-bath and then plunging suddenly into the river. I have seen the bones of a whole village occupying one common grave! It is as fatal among them as cholera. *The Indians say, and some white men, who had been there a number of years, concur with them, that they were strangers to the disease till the whites came there to inhabit. And one or two of the oldest white settlers, men who have lived there thirty years or more, attribute its appearance, without hesitation, to the turning up of the soil for agricultural purposes. They say, that in any of their river regions, the sticking of a plough or hoe into the ground is followed by ague, at certain seasons of the year, as invariably as the thunder-clap succeeds a flash of lightning.* These are the statements of people who are, of course, entirely ignorant of the principles of medicine or philosophy; but in a matter of mere fact, their observation is, probably, as much to be relied upon, as that of persons of much more cultivated minds, especially when we consider, that they can have no theory to support, and are consequently free from the very strong prejudices and fallacies of reasoning, which so frequently arise from this source. What staggers my belief on this subject, however, is, that the quantity of cultivated ground seems to me to be too small to produce such direful and wide spread effects, the country being very sparsely inhabited by whites, and the aborigines not cultivating it at all.

If what I have related possesses any interest, I hope that circumstance may serve as an apology for my having troubled you so much. With great respect, I am, Sir, your very obt. servant,

J. S. Whittle,
Asst. Surgeon, U. S. Navy.

To Professor Robley Dunglison.

—

*Philadelphia, June* 28, 1843.

Dear Sir,—The tarro (a species of arum) the roots of which are extensively used as food by nearly all the Polynesians, requires to be cultivated in shallow fresh water; and where natural marshes do not occur, they are constructed artificially by the natives. In such situations, it is common to find their towns, on all Islands within the tropics, in the Pacific ocean, standing in the midst of, or surrounded by "tarro patches;" suffering from musquitoes, and sometimes from the stench of stagnant water.

Such places were visited by the officers and scientific corps of the late Exploring Expedition, at the Friendly, Society, Fejee, Samoan, and Sandwich groups of Islands, without having in a single instance, to my knowledge, met with a case of intermitting fever, either amongst the natives or foreigners; or having suffered from the marshy atmosphere ourselves, although we were frequently exposed to it both day and night; most commonly we suffered from the violent heats of the tropical sun all day, on our shore journeys, and slept in open huts in the vicinity of the tarro patches at night.

On the arrival of the Expedition on our North West coast, I was one of a party who started immediately to the interior, and remained on the Willamet river about one month, at a place near which there was *no marshy ground*, that could lead us to suppose the "intermittents," by which we ALL suffered, could have their origin in them. Both the earth, and atmosphere were remarkably dry.

About the first of June, last year, I, with several of my voyaging companions, was attacked with intermitting fever, while crossing the equator, about midway in the Atlantic ocean, in the voyage home on board the U. S. ship Vincennes.

From the above observations, connected with previous suffering from the disease, I was led to make the remark to you, that the causes of intermitting fever, must be sought for in other sources than marshy exhalations.

If my humble experience can in any way prove useful, it will afford great pleasure to

Your obedient servant,
T. R. Peale.
Zoologist to the Exploring Expedition.

To Prof. R. Dunglison.

—

*Extract of a letter, dated Fort Macomb, Middle Florida, Jan.* 11, 1842, *from Dr. R. S. Holmes, U. S. A., to Dr. James R. Speer, of Pittsburgh.*

"My post, of which I have the sole medical command has been long known as the most unhealthy in Florida; so much so that it is always abandoned about the first of May, and not reoccupied until September. What influence makes it bear such a character, I cannot imagine. A full and rapid stream, of about one hundred yards in width, called the Swanne, flows by it. This river is navigable for steamboats fifty miles above this; is very deep; has not a single weed or decayed vegetable in it, and flows at the rate of three miles an hour; its banks are formed of light granular disintegrating limestone; the country around is dry, and thickly covered by a profuse growth of tall pines: there is not a marsh within five miles of us, and even then one that scarce deserves such a title; there is not a greater undergrowth of vegetable matter than I have seen about the most healthy posts in Florida, and the dry sand, which covers the whole country like a mantle, absorbs water like a sponge. I am inclined to think the geological nature of the banks of the river has something to do with the malaria, so prevalent here. I can conceive of nothing else: the situation is high, and a perfectly dry one, yet I have scarcely ever less than twenty on my sick list from sixty-six men; probably twenty more in the company are so debilitated with disease that they are unfit for active duty in the field."

